# Association between preconception folic acid intake and behavioural problems in children aged 2–8 years: a prospective cohort study

**DOI:** 10.1007/s00394-026-04094-5

**Published:** 2026-08-27

**Authors:** Alemayehu G. Mekonnen, Dereje G. Gete, Jenny Doust, Gita D. Mishra

**Affiliations:** Australian Women and Girls’ Health Research Centre, School of Public Health, Faculty of Health, Medicine and Behavioural Science, The University of Queensland, 4072, Brisbane, QLD Australia

**Keywords:** Preconception health, Folic acid supplementation, Behavioural problems, Cohort study

## Abstract

**Purpose:**

While maternal folic acid intake during periconception is well-known for preventing neural tube defects, its association with childhood behavioural outcomes remains understudied. This study examined the association between preconception folic acid intake and behavioural problems in children aged 2–8 years.

**Methods:**

We analysed data from 646 children and their 563 mothers participating in the Mothers’ and their Children’s Healthcare Experience Study, a sub-study of the 1989–95 cohort of the Australian Longitudinal Study on Women’s Health. Mothers reported preconception folic acid intake and rated their child’s behaviours using the Strengths and Difficulties Questionnaire. Analyses were conducted using Poisson regression with robust standard error.

**Results:**

Nearly two-thirds (63%) of mothers reported taking FA supplements prior to the child’s birth. Overall, 64% of children were reported to experience at least one behavioural problem across the behaviour difficulties subscales. After adjusting for potential confounders, children of mothers who reported taking FA supplements before conception had a 34% lower risk of peer relationship problems than those whose mothers did not (RR = 0.66; 95% CI 0.49–0.87). No associations were found between preconception folic acid intake and the total behavioural difficulties, emotional, conduct, or hyperactivity problem subscales.

**Conclusions:**

While large prospective studies are required to confirm these associations, our findings suggest that preconception folic acid intake may lower the risk of peer relationship problems in offspring. Supporting mothers to become more aware of the benefits of folic acid intake may contribute to optimal peer relationship outcomes in their offspring.

**Supplementary Information:**

The online version contains supplementary material available at https://doi.org/10.1007/s00394-026-04094-5.

## Introduction

Behavioural problems are common in childhood and affect approximately 10% to 20% of children worldwide [1]. In Australia, an estimated one in ten children (10–13%) experienced total behavioural difficulties between the ages of 5 and 12 years [2, 3], with relatively higher rates (39%) reported among younger children, particularly by age 3 years [2]. These childhood behavioural problems place a substantial burden on healthcare and child protection systems and are linked to poor academic performance and psychosocial functioning [4]. Given that behavioural problems in early life are linked with mental health disorders in later life, these issues have been the focus of attention even before conception [5, 6].

Apart from genetic factors, maternal intake of micronutrients before conception plays a crucial role in neurological development and optimal brain function [7]. Folic acid (FA), a synthetic form of folate, is an important micronutrient in several foetal physiological processes [8]. It plays a vital role in deoxyribonucleic acid methylation, which is involved in neurogenesis and neuronal differentiation [9]. The primary natural dietary sources of folate include green leafy vegetables, fruits, legumes, liver, and eggs [10]. As it is difficult for pregnant women to obtain adequate folate from diet alone, both national and international practice guidelines recommend that all women trying to conceive should take 400 µg of FA daily, starting at least three months before conception and continuing through early pregnancy [11, 12].

Offspring brain development and function may require different amounts of folate across distinct prenatal periods [13]. It has been proven that adequate FA intake during the periconception period prevents both the primary occurrence and recurrence of neural tube defects [9, 13]. Beyond neural tube closure, FA intake in the later stages of pregnancy also plays an important role in ongoing foetal brain development [14]. A growing body of evidence, including a follow-up study from a randomised controlled trial, suggests that continued FA supplementation during pregnancy is associated with enhanced cognitive performance in children [15]. Observational studies have further indicated that FA intake in the later stages of pregnancy is associated with improved cognitive outcomes in children [14, 16], including a reduced risk of autism spectrum disorder [17] and attention-deficit or hyperactivity disorder in offspring [18].

While existing studies have supported an association between maternal FA intake at various stages of pregnancy and cognitive and behavioural outcomes in children, their findings have been mixed and remain inconclusive [16, 17, 19, 20], possibly due to differences in the timing of folate exposure and outcome measures. For instance, Yang et al. reported an association between maternal FA supplementation in the second and third trimesters and neurobehavioral development in offspring [21], whereas Qiang et al. found an inverse association with cognitive development [16]. The other two studies found no association between maternal folate levels during pregnancy and children’s behavioural problems [19] or neurocognitive performance at 7 years of age [20]. Notably, most existing studies have focused on the association between FA intake during early and late pregnancy and offspring behavioural problems, with limited consideration of FA intake during the preconception period. By the end of the first trimester, major organogenesis is complete, and the brain is being programmed [8]; thus, adequate folate status before pregnancy may be particularly important. This study aimed to examine the association between preconception FA intake and behavioural difficulties in children aged 2–8 years, a period marked by rapid cognitive functions and emerging behavioural regulation [22].

## Methods

### Study design and participants

This study used data from the Mothers’ and their Children’s Healthcare Experience Study (MatCHES), a sub-study of the 1989–95 cohort of the Australian Longitudinal Study on Women's Health (ALSWH). The ALSWH is an ongoing population-based prospective cohort study that investigates the health and well-being of Australian women, as well as their access to and use of health services. The details of the recruitment methods are published elsewhere [23]. The ALSWH study has ethics approval from the Human Research Ethics Committees of the University of Queensland and the University of Newcastle. All women provided informed consent.

The sample for the current study was drawn from a cohort of women born between 1989 and 1995. They were followed up annually from 2013 to 2017 (the first five surveys), with a sixth survey in 2019 and a seventh survey in 2023. Women who had given birth to at least one child after the 31st of December 2014 (the end of the baseline survey) and had completed the seventh survey were invited to participate in the MatCHES survey. These women were aged 18 to 23 years at baseline and 28 to 35 years at the time of the seventh survey. The third survey of the ALSWH 1989–95 cohort was well positioned to describe maternal characteristics, as this study included children born after 2015 (Survey 3).

**Fig. 1:**
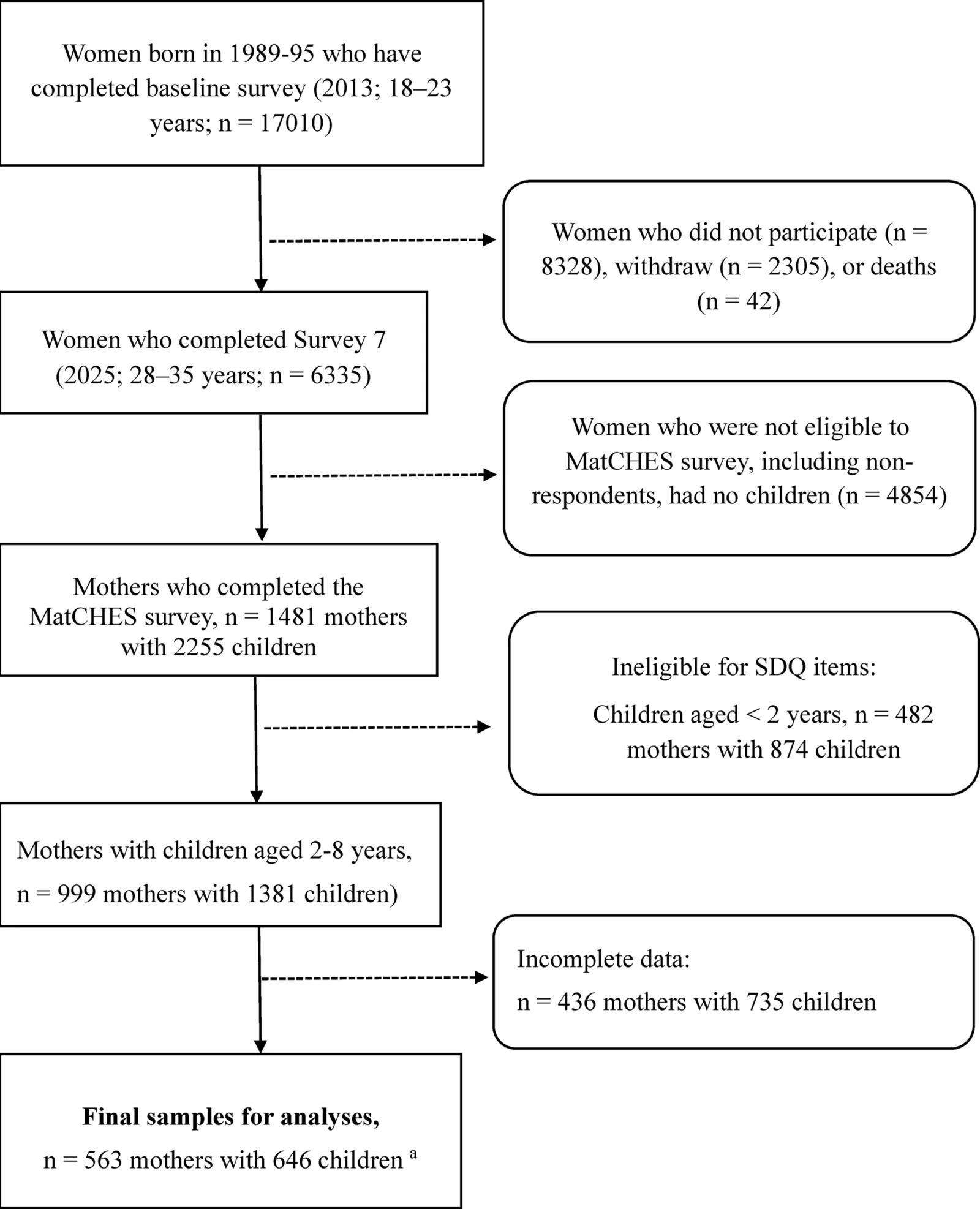
Flow diagram of sample selection for the analyses of preconception FA and behavioural problems in children aged 2–8 years. ^a^ Eight mothers participated with twins, while 58 mothers participated with two or more singleton children

Overall, 1481 mothers completed the MatCHES survey, providing data on 2,255 children and their own healthcare experiences, including FA intake in preparation for pregnancy. This study included only mothers whose children are eligible for the Strengths and Difficulties Questionnaire (SDQ) items (n = 999 mothers with 1381 children aged 2–8 years). Mothers with incomplete data on SDQ items were excluded, yielding a final analytic sample of 563 mothers with their 646 children. Eight mothers participated with twins, while 58 mothers participated with two or more singleton children (Fig. 1).

### Assessments of child behavioural problems

Child behavioural problems were assessed using the SDQ, a validated psychometric instrument widely used in both clinical and research settings [24]. Mothers were asked to rate their child’s behaviour over the past six months using a three-point scale: ranging from not true (0), somewhat true (1), and certainly true (2). The SDQ consists of 25 items with five sub-scales: emotional symptoms (fears, unhappy, worries, somatic, clingy), peer relationship problems (good friend, popular, best with adults, solitary, bullied), conduct problems (lies, fights, tempers, steals, obedient), hyperactivity/inattention problems (restless, distractible, fidgety, reflective, persistent), and prosocial behaviour (kindness, helping, caring, share, considerate), with each subscale scored from 0 to 10. The first four subscales are then combined to calculate a total difficulties score ranging from 0 to 40. Higher scores indicate greater behavioural difficulties, except for the prosocial behaviour scale, where lower scores indicate greater difficulties. In this study, each SDQ score was dichotomised into “normal” or "borderline/abnormal" based on binary cut-off scores recommended by Goodman: total behavioural difficulties (≥ 14), emotional symptoms (≥ 4), peer relationship problems (≥ 3), conduct problems (≥ 3), hyperactivity (≥ 6), and prosocial behaviours (≤ 5). This categorisation was used because SDQ subscale variables were skewed and could not be normalised, making analysis as continuous variables less appropriate. Furthermore, utilising Goodman's cut-offs provides a clinically meaningful interpretation of scores [24].

### Assessments of preconception FA intake

Preconception FA intake was assessed through maternal self-reports in the MatCHES survey. Mothers were asked the following question: “Before you became pregnant with [child’s name], did you do anything to improve your health in preparation for pregnancy?” One of the response options was “took folic acid”, which was recorded as yes or no. This question was adapted from existing tools to assess women’s pregnancy planning and preconception health behaviours [25]. While folate biomarkers were not measured in the present study, a validation study conducted in a multiethnic cohort of pregnant women demonstrated that self-reported FA supplement use was strongly correlated with blood folate concentrations [26], supporting the validity of self-reported FA intake measures.

### Assessments of maternal and child characteristics

Data on maternal age at child’s birth (continuous variable) were obtained from the MatCHES study. Other maternal characteristics were drawn from the ALSWH Survey 3 of 1989–95 cohort, as mothers who completed the MatCHES survey had given their first birth after 2015 (Survey 3). These include area of residence (categorised into major cities, regional/remote/very remote) based on a measure of relative access to service centres (e.g., health care, education, retail, and transportation) [27], marital status (categorised as married, de Facto/separated, or never married), highest level of education (≤ 12 years of schooling, certificates/diplomas, and university degree or higher), and ability to manage on income (categorised as impossible/difficult always, sometimes difficult, and easy/not too bad). Pre-pregnancy body mass index (BMI) was categorised as healthy weight (BMI < 25 kg/m^2^), overweight (25 to 30 kg/m^2^), and obesity (BMI ≥ 30 kg/m^2^). The underweight category was merged with the healthy weight category due to small numbers in the group (n = 18, 3.5%).

Intimate partner violence was assessed by the question "Have you ever been in a violent relationship with a partner?" with response options: no or yes. Perceived psychological stress was assessed using the Kessler Psychological Distress 10-item Scale (K10), a validated tool employed in national surveys in Australia [28]. Experience of gestational diabetes, hypertension, and pre-eclampsia was obtained with the question ‘Were you diagnosed with or treated for (a) gestational diabetes, (b) hypertension (high blood pressure) during pregnancy, and (c) pre-eclampsia during pregnancy? Women’s responses were recorded as yes or never. Pregnancy intention was obtained by asking mothers, “Just before you became pregnant, which most applies to you?” with responses recoded into two categories: intended to become pregnant, and intention kept changing or not indicated.

Maternal health behaviour factors were drawn from maternal responses for each live birth in the MatCHES survey. Data from Survey 3 of the ALSWH 1989–95 cohort were used for supplementary validation to assess the consistency of reported behaviours over time. Maternal fruit and vegetable intake was categorised based on the Australian Dietary Guidelines [29]. Physical activity was categorised as inactive/low, or active (moderate/high) according to the Australian Physical Activity Guidelines [30]. Smoking status was determined using self-reported smoking frequency and quantity from the ALSWH Survey 3 (1989–95 cohort), along with mothers’ responses in the MatCHES survey on whether they stopped or cut down smoking tobacco in preparation for pregnancy. Responses were categorised as: never smoked, ex-smokers, and current smokers. Mothers who reported cutting down on smoking tobacco in preparation for pregnancy were categorised with current smokers, as their newborns were still likely exposed to nicotine metabolites during the preconception period. Alcohol intake was determined using self-reported items on how often and how much alcohol was consumed per week from Survey 3 and mothers’ responses regarding whether they stopped or reduced alcohol intake in preparation for pregnancy. Responses were categorised as: non-drinkers, stopped drinking for pregnancy preparation, and any level of drinkers (including low-risk/high-risk drinkers, and those who cut down drinking), in line with recommendations for alcohol abstinence among women planning pregnancy [31]. Mothers were asked to report their children’s date of birth, sex recorded at birth, number of weeks of gestation at birth and child daycare arrangements in the MatCHES survey (Supplementary Table S1).

### Statistical analyses

Pearson's chi-square tests for categorical variables and Kruskal–Wallis tests for continuous variables were used to compare maternal and child characteristics in relation to child behavioural problems. Poisson regression with robust standard errors was applied to examine the association between preconception FA intake and each of the child behavioural outcomes. This method is reliable for prospective studies with binary outcomes, as it provides estimates of relative risks (RRs) with 95% confidence intervals (CIs), even in relatively small samples [32]. To account for the non-independence of observations due to mothers with multiple children, including twins, standard errors were clustered at the maternal level. In each model, children with normal behaviours were chosen as the reference group. Several sensitivity analyses were conducted to assess the robustness of our findings, including the use of alternative variable categorisations. Missing data on the outcome variables were excluded, whereas missing covariate and confounder data from Survey 3 of the ALSWH 1989–95 cohort were addressed using the last observation carried forward approach.

We also used a directed acyclic graph (DAG) to identify the minimally sufficient adjustment set for controlling potential confounders [33] (Supplementary Figure S1). Based on the DAG and relevant literature, models were adjusted for potential confounding factors and covariates. The first model was unadjusted. The second model was adjusted for maternal characteristics (age at child’s birth, area of residence, level of education, marital status, ability to manage on income, perceived psychological distress, intimate partner violence and number of children). The third model was additionally adjusted for pre-pregnancy BMI, maternal vegetable intake, physical activity, smoking, alcohol intake, child sex and age. All analyses were performed using Stata software (version 18.0; Stata-Corp).

## Results

This study included 646 children aged 2–8 years and their 563 mothers (Fig. 1). The median maternal age at the time of the child’s birth was 28 years. When comparing mothers with complete and incomplete outcome data, there were few differences in maternal sociodemographic and health behaviour characteristics. Mothers included in the analysis were more likely to have a university or higher degree, to have never been married, and to be primiparous. They were also more likely to report FA intake, to be physically active, and to stop drinking alcohol in preparation for pregnancy (Table S1).

In this study, nearly two-thirds (63%) of mothers reported taking FA supplements prior to the child’s birth. Mothers who reported taking FA prior to conception were more likely to live in major cities, have higher educational attainment, and be never married. They were also more likely to stop drinking alcohol in preparation for pregnancy, have intended to become pregnant, and have discussed the importance of FA intake with a general practitioner prior to the birth of a child (Table S2).

Table 1 presents the proportion of children with borderline/abnormal behavioural problems, stratified by child age and sex. Overall, 64% of children were reported to experience at least one behavioural problem across the total behavioural difficulties subscales. Girls were more likely than boys to experience emotional symptoms, particularly at age 3 years (p = 0.02). Hyperactivity or inattention and prosocial problems were more common among boys than girls (p < 0.05) (Table 1, Table S3). The mean (Standard Deviation) score of total difficulties was 10.4 (5.6), with 9.9 (4.8) at age 2 and 11.6 (7.5) among children aged 4–8 years (Table S4).

**Table 1:** Borderline/abnormal behavioural problems, stratified by child age and sex

Behavioural problems	Child sex	2 years	3 years	4–8 years	All
Total behavioural difficulties (n, %)	Boys	35 (25.4)	29 (22.1)	27 (40.9)	91 (27.2)
	Girls	22 (16.2)	31 (28.2)	21 (33.3)	74 (23.5)
	Total	57 (20.6)	60 (24.9)	48 (37.2)	165 (25.5)
Emotional symptoms (n, %)	Boys	8 (5.8)	15 (11.4)	18 (27.3)	41 (12.2)
	Girls	14 (10.3)	23 (20.9)	26 (41.3)	63 (20.4)
	Total	22 (8.0)	38 (15.8)	44 (34.1)	104 (16.1)
Peer relationship problems (n, %)	Boys	41 (29.7)	31 (23.7)	19 (28.8)	91 (27.2)
	Girls	41 (29.7)	24 (21.8)	14 (22.2)	78 (25.2)
	Total	82 (29.7)	55 (22.8)	33 (25.6)	137 (26.5)
Conduct problems (n, %)	Boys	48 (34.8)	58 (44.3)	28 (42.4)	134 (40.0)
	Girls	46 (33.8)	51 (46.4)	18 (28.6)	115 (37.2)
	Total	94 (34.1)	109 (45.2)	46 (35.7)	247 (38.5)
Hyperactivity or inattention (n, %)	Boys	55 (39.9)	36 (42.7)	33 (50.1)	144 (43.0)
	Girls	45 (33.1)	34 (30.9)	19 (30.2)	98 (31.7)
	Total	100 (36.2)	90 (37.3)	52 (40.3)	242 (37.5)
Prosocial behaviours (n, %)	Boys	44 (31.9)	31 (23.7)	19 (28.8)	94 (28.1)
	Girls	18 (13.2)	12 (10.9)	5 (7.9)	35 (11.3)
	Total	62 (22.5)	43 (17.8)	24 (18.6)	129 (20.0)
At least one behavioural problem across total difficulties subscales	Boys	86 (62.3)	84 (64.1)	46 (69.7)	216 (64.5)
	Girls	91 (66.9)	71 (64.5)	36 (57.1)	198 (64.1)
	Total	178 (64.5)	155 (64.3)	82 (63.6)	415 (64.2)

The total difficulties score ranges 0–40, and each of the four subscales ranges 0–10. Higher scores indicate greater behavioural difficulties, except for the prosocial behaviour scale, where lower scores indicate greater difficulties. Each SDQ score was categorised as either "normal" or "borderline/abnormal" based on binary cut-off scores recommended by Goodman: total behavioural difficulties (≥ 14), emotional symptoms (≥ 4), peer relationship behaviours (≥ 3), conduct (≥ 3), hyperactivity (≥ 6), and prosocial problems (≤ 5)

When examining the distribution of children’s behavioural problems according to maternal sociodemographic characteristics, hyperactivity problems were more common among children of mothers with lower educational attainment and those who reported difficulty on the ability to manage on an income. Children who experienced total behavioural difficulties and problems across all four subscales were more likely to be born to mothers who reported higher psychological distress and who were overweight or obese before conception. Peer relationship problems were more common among children whose mothers did not take FA prior to conception and among those whose mothers reported any alcohol consumption prior to the child’s birth (Table 2).

**Table 2:** Maternal and child characteristics according to borderline/abnormal behavioural problems in children aged 2–8 years (n = 646)^a^

Characteristics	Total behavioural difficulties (n = 165; 25.5%)	Emotional sympto ms (n = 104; 16.1%)	Peer relationship problem (n = 170; 26.3%)	Conduct problem (n = 249; 38.5%)	Hyperactivity/ inattention (n = 242; 37.5%)	Prosocial problems (n = 105; 20.3%)
Maternal age at child’s birth in years, median (Inter Quartile Range)	28 (26,30)	27 (25,29)	29 (27,30)	28 (27,30)	28 (26,30)	29 (27,30)
*Area of residence*						
Major cities	103 (25.2)	60 (14.7)	114 (27.9)	151 (36.9)	148 (36.2)	78 (19.1)
Regional/Remote	62 (26.2)	44 (18.6)	56 (23.6)	98 (41.4)	94 (39.7)	51 (21.5)
P-value	0.78	0.19	0.23	0.26	0.37	0.45
*Highest level of education*						
≤12 years of schooling	36 (24.0)	20 (13.3)	44 (29.3)	61 (40.8)	60 (40.0)	32 (21.3)
Certificates/diploma	49 (30.2)	28 (17.3)	40 (24.7)	72 (44.4)	76 (46.9)	26 (16.1)
University degree/higher	80 (24.0)	56 (16.8)	86 (25.8)	116 (34.7)	106 (31.8)	71 (21.3)
P-value	0.42	0.72	0.72	0.15	0.01	0.50
*Marital status*						
Married	25 (25.8)	21 (21.6)	30 (30.9)	33 (34.0)	30 (30.9)	17 (17.5)
De Facto/separated	56 (25.7)	28 (12.8)	49 (22.5)	90 (41.3)	91 (41.7)	50 (22.9)
Never married	84 (25.4)	55 (16.7)	91 (27.6)	126 (38.2)	121 (36.7)	62 (18.8)
P-value	0.95	0.24	0.34	0.53	0.24	0.55
*Ability to manage on income*						
Impossible/difficult always	38 (40.8)	21 (22.6)	32 (34.4)	53 (57.0)	50 (53.8)	20 (21.5)
Sometimes difficult	61 (26.0)	39 (16.6)	64 (27.2)	88 (37.4)	88 (37.4)	44 (18.2)
Easy/not too bad	66 (20.8)	44 (13.9)	74 (23.3)	108 (34.1)	104 (32.8)	65 (20.5)
P-value	0.01	0.23	0.16	0.01	0.03	0.88
*Ever been in a violent relationship with partner*						
Yes	39 (38.2)	23 (22.5)	33 (33.4)	51 (50.0)	46 (45.1)	21 (20.6)
No	111 (22.5)	76 (15.4)	117 (23.7)	182 (36.9)	178 (36.1)	97 (19.7)
P-value	0.01	0.15	0.13	0.06	0.01	0.62
*Perceived psychological stress*						
Low psychological distress	34 (17.4)	24 (12.2)	41 (20.9)	56 (28.6)	57 (29.1)	39 (19.9)
Moderate distress	40 (22.2)	26 (14.4)	50 (27.8)	66 (36.7)	63 (35.0)	36 (20.0)
Severe distress	52 (29.4)	31 (17.5)	47 (26.5)	82 (46.3)	72 (40.7)	38 (21.5)
Very high distress	39 (41.1)	23 (24.7)	32 (34.4)	45 (48.4)	50 (53.7)	16 (17.2)
P-value	< 0.01	0.05	0.06	< 0.01	<0.01	0.87
*Pre-pregnancy Body Mass Index *^*b*^						
Healthy weight (< 25 kg/m^2^)	78 (21.3)	48 (13.1)	81 (22.1)	131 (35.7)	122 (33.3)	70 (19.1)
Overweight (25 to 30 kg/m^2^)	45 (29.2)	30 (19.5)	48 (31.2)	55 (35.7)	72 (46.7)	25 (16.2)
Obesity (≥ 30 kg/m^2^)	41 (34.2)	25 (20.8)	40 (33.3)	61 (50.8)	47 (39.2)	32 (26.7)
P-value	0.02	0.12	0.04	0.02	0.03	0.10
*Number of children per mother*						
One	86 (24.6)	40 (11.5)	104 (29.8)	138 (39.5)	136 (39.0)	65 (18.6)
Two and more	79 (26.6)	64 (21.6)	66 (22.2)	111 (37.4)	106 (35.7)	64 (21.6)
P-value	0.57	0.01	0.02	0.57	0.39	0.35
*Pregnancy intention*						
Intended to get pregnant	119 (23.2)	77 (15.0)	123 (24.0)	178 (34.7)	177 (34.5)	106 (20.7)
Intention kept changing/not expressed	46 (34.8)	27 (20.4)	47 (35.6)	71 (53.8)	65 (49.2)	23 (17.4)
P-value	0.02	0.29	0.02	<0.01	0.01	0.62
*Gestational diabetes mellitus*						
Yes	24 (30.4)	14 (17.7)	22 (27.8)	31 (39.2)	28 (35.4)	20 (25.5)
No	141 (24.9)	90 (15.8)	148 (26.1)	218 (38.4)	214 (37.7)	109 (19.2)
P-value	0.29	0.67	0.74	0.89	0.69	0.20
*Hypertension during pregnancy or preeclampsia*						
Yes	23 (31.5)	10 (13.7)	18 (24.7)	28 (38.4)	32 (43.8)	17 (23.3)
No	142 (24.8)	94 (16.4)	152 (26.5)	221 (38.6)	210 (36.6)	112 (19.6)
P-value	0.21	0.55	0.73	0.97	0.23	0.60
**Maternal preconception behaviour factors**						
*Intake of FA supplements before conception*						
No	61 (26.2)	45 (19.3)	72 (30.1)	102 (43.8)	97 (41.6)	52 (22.3)
Yes	102 (25.2)	57 (14.1)	97 (24.0)	145 (35.9)	143 (35.4)	77 (19.1)
P-value	0.94	0.20	0.09	0.08	0.18	0.19
*Fresh fruit intake (serves per day)*						
< 2 serves of fruit per day	104 (26.1)	60 (15.1)	105 (26.4)	154 (38.7)	162 (40.7)	86 (21.6)
≥2 serves of fruit per day	48 (22.7)	32 (15.2)	55 (26.1)	78 (37.0)	65 (30.8)	39 (18.5)
p-value	0.25	0.90	0.99	0.58	0.23	0.69
*Fresh vegetable intake (serves per day)*						
None/ ≤ 1 serves per day	38 (30.6)	21 (10.8)	40 (32.3)	54 (43.6)	54 (43.6)	24 (19.4)
2 serves per day	49 (27.5)	23 (12.9)	43 (24.2)	69 (38.7)	76 (42.7)	34 (19.1)
3 serves per day	34 (20.1)	27 (16.0)	41 (24.3)	58 (34.3)	55 (32.5)	39 (23.1)
4 serves per day	20 (23.0)	13 (14.4)	27 (31.0)	33 (37.9)	26 (29.9)	21 (24.1)
≥5 serves per day	11 (21.6)	8 (15.7)	9 (17.6)	18 (35.3)	16 (31.4)	7 (13.7)
p-value	0.21	0.11	0.30	0.59	0.11	0.38
*Physical activity*						
Inactive/low	56 (25.2)	38 (17.1)	47 (21.2)	95 (42.8)	89 (40.1)	46 (20.7)
Moderate/high	109 (25.7)	66 (15.6)	123 (29.0)	154 (36.3)	153 (36.1)	83 (19.6)
P-value	0.89	0.61	0 0.03	0.11	0.31	0.72
*Smoking status *^*c*^						
Never smoked	111 (23.4)	75 (15.8)	124 (26.2)	173 (36.5)	168 (35.4)	93 (19.6)
Ex-smoker/stopped smoking	21 (33.8)	14 (22.6)	18 (29.0)	25 (40.3)	24 (38.7)	7 (11.3)
Current smoker	33 (30.0)	15 (13.6)	28 (29.0)	51 (46.4)	50 (45.4)	29 (26.4)
p-value	0.10	0.29	0.86	0.15	0.14	0.06
*Alcohol consumption status *^*c*^						
Non-drinker	22 (32.1)	16 (23.5)	23 (33.8)	29 (42.6)	30 (44.1)	11 (16.2)
Stopped drinking	41 (30.6)	22 (16.4)	46 (34.3)	48 (35.8)	48 (35.8)	29 (21.6)
Low risk/high risk drinker	102 (23.0)	66 (14.9)	101 (22.7)	172 (38.7)	164 (36.9)	89 (22.3)
P-value	0.08	0.31	0.01	0.63	0.47	0.65
**Children’s characteristics**						
*Sex recorded at birth*						
Boys	91 (27.2)	41 (12.2)	91 (27.2)	134 (40.0)	144 (43.0)	94 (28.1)
Girls	74 (23.9)	63 (20.4)	78 (25.2)	115 (37.2)	98 (31.7)	35 (11.3)
P-value	0.45	0.02	0.64	0.41	0.03	<0.01
*Gestational age in completed weeks *^*b*^						
Pre-term birth (< 37 weeks)	17 (36.2)	8 (17.0)	18 (38.3)	18 (38.3)	22 (46.8)	16 (34.0)
Term birth (37–42 weeks)	146 (24.7)	96 (16.2)	148 (25.0)	229 (38.7)	218 (36.8)	110 (18.6)
P-value	0.22	0.50	0.03	0.86	0.35	0.01
*Child day care arrangements*						
No	75 (28.1)	54 (20.2)	74 (27.7)	106 (38.6)	103 (38.6)	52 (19.5)
Yes (1–5 days per week)	90 (24.1)	50 (13.4)	95 (25.4)	143 (38.2)	137 (36.6)	76 (20.3)
P-value	0.21	0.56	0.76	0.87	0.87	0.96

^a^ P-values are from the Pearson chi-square test. ^b^ Variable categories merged because of the small number of women in the group: underweight (n = 18, 3.5%) with normal-weight, and post-term births (n = 17, 3.3%) with term births. ^c^ Mothers who reported cutting down on smoking or alcohol intake in preparation for pregnancy were merged with current smokers and high-risk drinker, respectively, as their newborns were still partially exposed to these substances during the preconception period

Table 3 presents the relationship between preconception FA intake and the risk of behavioural problems in children aged 2–8 years. After adjusting for potential confounders and covariates, children of mothers who reported taking FA supplements before conception had a 34% lower risk of peer relationship problems than those whose mothers did not (RR = 0.66; 95% CI 0.49–0.87). When the adjustment was made step by step, the inclusion of maternal vegetable intake and alcohol consumption strengthened the association, suggesting that these maternal factors may have confounded the crude relationship. However, no associations were found between preconception FA intake and the total behavioural difficulties, emotional, conduct, or hyperactivity problem subscales (Table 3, Supplementary Figure S2). We also examined the association between preconception FA intake and the risk of behavioural problems across two developmental periods (2–3 years and 4–8 years) and child sex. The associations between FA intake and peer relationship problems were more evident among children aged 2–3 years and among boys. We found no association between FA intake and behavioural problems among children aged 4–8 years and among girls (Table S5).

**Table 3:** Association between preconception FA intake and borderline/abnormal behavioural problems in children aged 2–8 years, (RR (95% CI)^a^

		Total behavioural difficulties	Emotional problems	Peer relationship problems	Conduct problems	Hyperactivity/ inattention	Prosocial behaviours
Preconception FA intake (*Ref.* = *No*)	Model 1	0.96 (0.73, 1.27)	0.73 (0.50, 1.06)	0.77 (0.59, 1.00)	0.82 (0.67, 1.00)	0.85 (0.69, 1.04)	0.85 (0.62, 1.17)
	Model 2	1.12 (0.83, 1.51)	0.85 (0.59, 1.24)	0.76 (0.58, 1.00)	0.89 (0.72, 1.09)	0.95 (0.77, 1.16)	0.81 (0.58, 1.13)
	Model 3	1.07 (0.77, 1.48)	0.80 (0.53, 1.20)	**0.66 (0.49, 0.87)**	0.95 (0.75, 1.21)	0.98 (0.79, 1.21)	0.75 (0.53, 1.08)

^a^ Relative risk (RR) with 95% confidence intervals (CI) were estimated using Poisson regression with robust standard error variance (0 = Normal, 1 = Borderline/abnormal behaviours), with "Normal behaviours" as the reference group. **Model 1** was crude relative risks with 95% confidence interval. **Model 2** adjusted for maternal characteristics (maternal age at child’s birth, area of residence, level of education, marital status, ability to manage on income, number of children, perceived psychological distress, and intimate partner violence). **Model 3** additionally adjusted for pre-pregnancy BMI, maternal vegetable intake, physical activity, smoking, alcohol intake, child sex and age. Bold estimates indicate statistically significant associations (p < 0.05)

## Discussion

In this population-based prospective cohort study, we found that maternal preconception FA intake was associated with a lower risk of peer relationship problems in the offspring. No significant associations were observed between preconception FA intake and the total behavioural difficulties, emotional, conduct, or hyperactivity problem subscales.

Previous studies examining the association between maternal folate and childhood behavioural problems have reported inconsistent findings, leaving the relationship inconclusive. For instance, a recent meta-analysis found that prenatal FA supplementation was associated with a lower risk of total behavioural problems in children [14]. In contrast, two studies found no association between maternal FA intake and child behavioural outcomes overall [19, 20]. Findings from the Danish National Birth Cohort further indicated that periconceptional FA intake was associated with a lower risk of hyperactivity problems but a higher risk of emotional symptoms in children [34]. A recent cohort study has suggested that maternal FA supplementation during pregnancy was associated with an increased risk of externalising behavioural problems in offspring [16]. Consistent with our findings, Schlotz et al. reported a higher risk of peer problems among children whose mothers had low folate levels during early pregnancy [35]. These mixed findings suggest that maternal FA supplementation may not be uniformly associated with specific childhood behavioural outcomes due to differences in exposure timing and dosage [18]. Methodological differences may also contribute to discrepancies between our findings and prior studies, including variations in the timing of FA exposure, behavioural outcome measures, and adjustment for potential confounders. Furthermore, whereas many previous studies examined folate intake during pregnancy, our study focused on preconception FA intake, a distinct exposure window that may have different implications for offspring neurodevelopment [7].

While the origins of behavioural difficulties in offspring are multifaceted and the mechanisms are complex [7], several potential mechanisms may underlie the observed association between preconception FA intake and a lower risk of peer relationship problems in the current study. Beyond its well-established role in preventing neural tube defects, evidence from limited human research found the association between periconceptional FA intake and optimal cognitive performance and behavioural outcomes in offspring [21, 36]. FA serves as a substrate for numerous enzymatic reactions involved in neurogenesis and neuronal differentiation, thereby contributing to optimal foetal brain development and function [14].

Another possible explanation could be its role in reducing prenatal oxidative stress (an excessive production of free radicals mainly generated during saturated fat metabolism) [9]. As a coenzyme in one-carbon metabolic reactions, FA enhances antioxidant capacity by increasing serum concentration of glutathione, a key molecule that neutralises reactive oxygen species, and by lowering homocysteine concentrations [37]. Thus, FA supplementation prior to conception and during early pregnancy, through its role in the methylation process and as a cofactor for several enzymatic reactions, may help maintain the development of social cognition and emotional regulation, ultimately reducing the risk of peer relationship problems in early childhood [14, 36].

While our analyses indicate an association between preconception FA intake and a lower risk of peer relationship problems in offspring, this relationship appeared stronger after adjusting for maternal vegetable intake and alcohol consumption. Although there is no evidence for an association with peer problems, the link between maternal vegetable intake, the main source of dietary folate [8], and children’s behavioural outcomes has been demonstrated in the literature [7, 38]. In our study, mothers who reported taking FA were also more likely to meet the recommended daily vegetable intake (75% vs. 58%; p = 0.01) and to stop alcohol intake (85% vs. 60%; p < 0.01), both of which are independently associated with optimal behavioural outcomes in children [38, 39]. These findings suggest that preconception FA supplementation occurs within a broader pattern of healthier preconception behaviour changes, which may also contribute to improved behavioural outcomes in offspring.

### Implications for clinical practice and public health policy

The observed association between maternal preconception FA intake and a lower risk of peer relationship problems in offspring highlights the potential importance of promoting FA supplementation before conception. Healthcare providers may need to initiate discussions about FA supplementation with women of reproductive age during routine primary care encounters rather than waiting until pregnancy is confirmed. Public health initiatives aimed at improving awareness and uptake of FA supplementation before conception may therefore be warranted, as adherence to FA recommendations may potentially contribute to better behavioural outcomes in offspring, including better peer relationships.

### Strengths and limitations

This study was based on a population-based cohort study design with detailed maternal and child health data, enabling comprehensive analyses with adjustment for relevant confounders. However, the following limitations should also be considered. First, child behavioural problems were assessed using maternal reports of SDQ items rather than clinician-led diagnostic assessments, which may have introduced reporting bias and random measurement error. Nevertheless, SDQ is a validated and reliable psychometric instrument for assessing children’s behavioural problems and has been widely used in both clinical and research settings [24, 40]. Second, preconception FA intake was also self-reported and may have been subject to recall and information bias. While a validation study has shown correlations between self-reported FA intake and serum folate levels [26], combining subjective questionnaires with objective biomarkers provides a more valid assessment of folate status [14] and warrants further research. In the present study, we were unable to implement such approaches due to the unavailability of clinical data linkage on FA intake before conception. Third, FA intake, as a single nutrient, may be confounded by broader maternal dietary patterns and limit the generalisability of our conclusions. However, we adjusted for maternal fruit and vegetable intake and performed sensitivity analyses. Finally, our sample size may have affected the statistical power of the analyses, and additional longitudinal studies with a large sample size are needed to confirm the observed associations and clarify the underlying mechanisms.

## Conclusions

While large prospective studies are required to confirm associations and the underlying biological mechanisms, our findings suggest that preconception FA intake may lower the risk of peer relationship problems in offspring. Supporting mothers to become more aware of the benefits of FA intake may contribute to optimal peer relationship outcomes in their offspring.

## Supplementary Information

Below is the link to the electronic supplementary material.

Supplementary Material 1.

## Data Availability

Application to access ALSWH data can be made via the portal (https:/alswh.org.au/for-data-users/applying-for-data).

## Abbreviations

ALSWH: Australian Longitudinal Study on Women's Health
BMI: Body mass index
CIs: Confidence intervals
FA: Folic acid
MatCHES: Mothers’ and their Children’s Healthcare Experience Study
RRs: Relative risks
SDQ: Strengths and Difficulties Questionnaire
