# Supplementary material for: Association between preconception folic acid intake and behavioural problems in children aged 2–8 years: a prospective cohort study
Source: Eur J Nutr. 2026 Aug 27;65(6):241. doi: 10.1007/s00394-026-04094-5 (PMC13522053; doi:10.1007/s00394-026-04094-5)
Supplement: Supplementary file 1 [file 394_2026_4094_MOESM1_ESM.docx]

**Supplementary Informations**

**Table S1:** Comparison of characteristics of mothers and children with complete and incomplete data on SDQ items (n = 1,381) ^a^.

| **Characteristics** | | Included in the analysis  (n=646;46.8%) | Incomplete data on SDQ items (n = 735; 53.2%) | P-value |
| --- | --- | --- | --- | --- |
| Maternal age at child’s birth in years, median (Inter Quartile Range) | | 28 (27,30) | 27 (25,28) | 0.10 |
| Area of residence | |  |  | 0.81 |
|  | Major cities | 409 (63.3) | 470 (63.9) |  |
|  | Regional/remote | 237 (36.7) | 265 (36.1) |  |
| Highest level of education | |  |  | 0.05 |
|  | ≤12 years of schooling | 150 (23.2) | 194 (26.4) |  |
|  | Certificates/diploma | 162 (25.1) | 217 (29.5) |  |
|  | University degree/higher | 333 (51.1) | 323 (44.0) |  |
| Marital status | |  |  | 0.01 |
|  | Married | 97 (15.1) | 205 (27.9) |  |
|  | De Facto/separated | 218 (33.7) | 231 (31.1) |  |
|  | Never married | 330 (51.1) | 298 (40.5) |  |
| Ability to manage on income | |  |  | 0.21 |
|  | Impossible/difficult always | 93 (14.4) | 137 (18.6) |  |
|  | Sometimes difficult | 235 (36.4) | 251 (34.2) |  |
|  | Easy/not too bad | 317 (49.1) | 346 (47.1) |  |
| Ever been in a violent relationship with a partner | | |  | 0.16 |
|  | Yes | 102 (15.8) | 105 (14.3) |  |
|  | No | 493 (76.3) | 594 (80.8) |  |
| Perceived psychological stress | |  |  | 0.56 |
|  | Low psychological distress | 196 (30.3) | 207 (28.2) |  |
|  | Moderate distress | 178 (27.5) | 218 (29.7) |  |
|  | Severe distress | 177 (27.4) | 188 (25.6) |  |
|  | Very high distress | 93 (14.4) | 121 (16.5) |  |
| Pre-pregnancy Body Mass Index | |  |  | 0.63 |
|  | Healthy weight (18.5 to <25 kg/m^2^) | 367 (57.3) | 428 (59.3) |  |
|  | Overweight (25 to 30 kg/m^2^) | 154 (24.0) | 158 (21.9) |  |
|  | Obesity (BMI ≥ 30 kg/m^2^) | 120 (18.7) | 136 (18.8) |  |
| Number of children per mother | |  |  | <0.01 |
|  | One | 310 (59.9) | 73 (47.4) |  |
|  | Two and more | 207 (40.1) | 81 (52.6) |  |
| Gestational diabetes mellitus | |  |  | 0.24 |
|  | Yes | 79 (12.2) | 75 (10.2) |  |
|  | No | 567 (87.8) | 656 (89.3) |  |
| Hypertension during pregnancy or preeclampsia | | |  | 0.17 |
|  | Yes | 73 (11.3) | 84 (11.4) |  |
|  | No | 573 (89.9) | 647 (88.0) |  |
|  | **Maternal preconception health factors** | | |  |
| Took folic acid | |  |  | 0.01 |
|  | Yes | 404 (63.4) | 391 (56.8) |  |
|  | No | 233 (36.6) | 297 (43.2) |  |
| Fresh fruit intake (serves per day) | |  |  | 0.01 |
|  | < 2 serves of fruit per day | 398 (65.4) | 504 (74.0) |  |
|  | ≥2 serves of fruit per day | 211 (34.6) | 177 (26.0) |  |
| Fresh vegetable intake (serves per day) | | |  | 0.37 |
|  | None/ ≤ 1 serves per day | 124 (65.0) | 134 (19.7) |  |
|  | 2 serves per day | 178 (29.2) | 222 (32.6) |  |
|  | 3 serves per day | 169 (27.8) | 199 (29.2) |  |
|  | 4 serves per day | 87 (14.3) | 78 (11.4) |  |
|  | ≥5 serves per day | 51 (8.4) | 48 (7.1) |  |
| Physical activity | |  |  | 0.01 |
|  | Inactive/low | 221 (34.2) | 310 (42.2) |  |
|  | Active (moderate/high) | 424 (65.6) | 424 (57.7) |  |
| Smoking status | |  |  | 0.48 |
|  | Never smoked | 474 (73.4) | 537 (73.1) |  |
|  | Ex-smoker/stopped smoking | 61 (9.4) | 86 (11.7) |  |
|  | Current smoker/cut down smoking | 110 (17.0) | 111 (15.1) |  |
| Alcohol intake | |  |  | 0.01 |
|  | Non-drinker | 68 (10.5) | 100 (13.6) |  |
|  | Stopped drinking in preparation for pregnancy | 134 (20.7) | 99 (13.5) |  |
|  | Low risk/high risk drinker | 444 (68.7) | 536 (72.9) |  |
| **Children’s characteristics** | |  |  |  |
| Child age | |  |  | <0.01 |
|  | At 2 years | 276 (53.4) | 71 (9.7) |  |
|  | At 3 years | 241 (37.3) | 83 (11.3) |  |
|  | At 4 years | 31 (4.8) | 165 (22.4) |  |
|  | 5-8 years | 98 (15.7) | 392 (53.3) |  |
| Child sex recorded at birth | |  |  | 0.69 |
|  | Boys | 335 (51.9) | 391 (53.2) |  |
|  | Girls | 309 (47.8) | 340 (46.3) |  |
| Gestational age in completed weeks | |  |  | 0.19 |
|  | Pre-term births (< 37 weeks) | 47 (7.3) | 66 (9.0) |  |
|  | Term birth (37-42 weeks) | 594 (91.6) | 631 (85.9) |  |
| Child day care arrangements | |  |  | <0.01 |
|  | No | 267 (41.3) | 496 (67.5) |  |
|  | Yes | 374 (57.9) | 132 (18.0) |  |

^a^ Values in the table are column percentages (%), and the numbers may not sum to the total due to missing data. Post-term births were merged with term births because of the small number of women in the group (n = 17, 3.3%). Mothers who reported cutting down on smoking or alcohol consumption in preparation for pregnancy were merged with current smokers and any-level drinkers, respectively, as their newborns were still partially exposed to these substances during the preconception period. P-values are from the Pearson chi-square test and Mann–Whitney test.

**Table S2:** Maternal characteristics according to preconception folic acid intake (n = 646) ^a^.

| **Characteristics** | | **Took folic acid supplement** | |  |
| --- | --- | --- | --- | --- |
|  | | Yes (n = 404) | No (n = 233) | P-value |
| Maternal age at child’s birth in years, median (Inter Quartile Range) | | 27 (26,29) | 28 (26,29) | 0.79 |
| Area of residence | |  |  | 0.01 |
|  | Major cities | 276 (67.5) | 126 (30.8) |  |
|  | Regional/Remote | 128 (54.0) | 107 (45.2) |  |
| Highest level of education | |  |  | 0.01 |
|  | ≤12 years of schooling | 82 (54.7) | 66 (44.0) |  |
|  | Certificates/diploma | 79 (48.8) | 82 (50.6) |  |
|  | University degree/higher | 243 (73.0) | 85 (25.5) |  |
| Marital status | |  |  | 0.01 |
|  | Married | 57 (58.8) | 40 (41.2) |  |
|  | De Facto/separated | 135 (61.9) | 81 (37.2) |  |
|  | Never married | 212 (64.2) | 112 (33.9) |  |
| Ability to manage on income | |  |  | 0.09 |
|  | Impossible/difficult always | 53 (57.0) | 37 (39.8) |  |
|  | Sometimes difficult | 138 (58.7) | 94 (40.0) |  |
|  | Easy/not too bad | 213 (67.2) | 102 (32.2) |  |
| Ever been in a violent relationship with partner | | | | 0.41 |
|  | Yes | 59 (57.8) | 42 (41.2) |  |
|  | No | 314 (63.7) | 174 (35.3) |  |
| Perceived psychological stress | |  |  | <0.01 |
|  | Low psychological distress | 139 (70.9) | 64 (27.6) |  |
|  | Moderate distress | 121 (68.0) | 56 (31.5) |  |
|  | Severe distress | 99 (55.9) | 76 (42.9) |  |
|  | Very high distress | 44 (47.3) | 47 (50.5) |  |
| Pre-pregnancy Body Mass Index | |  |  | 0.05 |
|  | Healthy weight (<25 kg/m^2^) | 241 (65.7) | 120 (32.2) |  |
|  | Overweight (25 to 30 kg/m^2^) | 98 (63.6) | 55 (35.7) |  |
|  | Obese (≥ 30 kg/m^2^) | 63 (52.5) | 56 (46.7) |  |
| Number of children per mother | |  |  | 0.44 |
|  | One | 221 (63.3) | 125 (35.8) |  |
|  | Two and more | 183 (61.6) | 108 (36.4) |  |
| Pregnancy intention | |  |  | <0.01 |
|  | Intended to become pregnant | 381 (74.3) | 130 (25.3) |  |
|  | Intention kept changing/not expressed | 23 (17.4) | 103 (78.0) |  |
| **Maternal health behaviours** | | | | |
| Fresh fruit intake (pieces per day) | | |  | 0.02 |
|  | < 2 pieces of fruit per day | 234 (59.5) | 159 (40.5) |  |
|  | ≥2 pieces of fruit per day | 149 (70.9) | 61 (29.1) |  |
| Fresh vegetable intake (serves per day) | | |  | 0.01 |
|  | None/ ≤ 1 serves per day | 63 (52.1) | 58 (47.9) |  |
|  | 2 serves per day | 109 (61.2) | 69 (38.7) |  |
|  | 3 serves per day | 109 (64.9) | 59 (35.1) |  |
|  | 4 serves per day | 65 (75.6) | 21 (24.4) |  |
|  | ≥5 serves per day | 37 (74.0) | 13 (26.0) |  |
| Physical activity | |  |  | 0.06 |
|  | Inactive/low | 127 (57.5) | 91 (39.1) |  |
|  | Moderate/high | 277 (65.3) | 142 (33.5) |  |
| Smoking status ^b^ | |  |  | 0.14 |
|  | Never smoked | 307 (64.8) | 163 (34.4) |  |
|  | Ex-smoker/stopped smoking | 38 (62.3) | 22 (36.1) |  |
|  | Current smoker/cut down smoking | 59 (53.6) | 48 (43.6) |  |
| Alcohol consumption status ^b^ | |  |  | **<0.01** |
|  | Non-drinker | 41 (60.3) | 27 (39.7) |  |
|  | Stopped drinking | 112 (85.6) | 21 (15.7) |  |
|  | Low risk/high risk | 251 (56.5) | 185 (41.7) |  |
| A general practitioner had discussed folic acid supplements before pregnancy | | | | <0.01 |
|  | Yes | 210 (80.5) | 49 (18.8) |  |
|  | No | 167 (50.2) | 160 (48.1) |  |

^a^ Values in the table are row percentages (%), and the numbers may not sum to total due to missing data (violent relationship with partner n= 51, intake of folic acid n= 9, BMI n = 4, fruit intake n = 37 vegetable intake n = 34). ^b^ Mothers who reported cutting down on smoking, alcohol consumption, recreational drug use in preparation for pregnancy were merged with current smokers, any-level drinkers and tried recreational drugs, respectively, as their newborns were still partially exposed to these substances during the preconception period. P-values are from the Pearson chi-square test and Mann–Whitney test.

**Table S3:** Strengths and Difficulties Questionnaire scores, stratified by child sex *(*n = 646) ^a^.

| Child behavioural problems | | All  (n = 646) | Boys  (n = 335) | Girls  (n = 309) | P-value |
| --- | --- | --- | --- | --- | --- |
|  |  | N (%) | N (%) | N (%) |  |
| Total behavioural difficulties | | | |  |  |
|  | Normal | 481 (74.5) | 244 (72.8) | 235 (76.1) | 0.46 |
|  | Borderline/abnormal | 165 (25.5) | 91 (27.2) | 74 (23.5) |  |
| Emotional symptoms | |  |  |  | **0.02** |
|  | Normal | 542 (83.9) | 294 (87.8) | 246 (79.6) |  |
|  | Borderline/abnormal | 104 (16.1) | 41 (12.2) | 63 (20.4) |  |
| Peer relationship problems | |  |  |  | 0.64 |
|  | Normal | 380 (73.5) | 244 (72.8) | 231 (74.8) |  |
|  | Borderline/abnormal | 137 (26.5) | 91 (27.2) | 78 (25.2) |  |
| Conduct problems | |  |  |  | 0.41 |
|  | Normal | 397 (61.5) | 201 (60.0) | 194 (62.8) |  |
|  | Borderline/abnormal | 247 (38.5) | 134 (40.0) | 115 (37.2) |  |
| Hyperactivity or inattention | |  |  |  | **0.01** |
|  | Normal | 404 (62.5) | 191 (57.0) | 211 (68.3) |  |
|  | Borderline/abnormal | 242 (37.5) | 144 (43.0) | 98 (31.7) |  |
| Prosocial behaviours | |  |  |  | **<0.01** |
|  | Normal | 517 (80.0) | 241 (71.9) | 274 (88.7) |  |
|  | Borderline/abnormal | 129 (20.0) | 94 (28.1) | 35 (11.3) |  |
| Number of behavioural problems across total difficulties subscales (emotional, conduct, hyperactivity, and peer problems) | | | | | 0.75 |
|  | No problem | 231 (35.6) | 119 (35.5) | 111 (35.9) |  |
|  | One | 183 (28.3) | 87 (26.0) | 95 (30.7) |  |
|  | Two | 141 (21.8) | 77 (23.0) | 64 (20.7) |  |
|  | Three | 64 (9.9) | 39 (11.6) | 25 (8.1) |  |
|  | Four | 27 (4.2) | 13 (3.9) | 14 (4.5) |  |

^a^ Values in the table are column percentages, obtained using Pearson’s chi-square test. The total difficulties score ranges 0–40, and each of the four subscales ranges 0–10. Higher scores indicate greater behavioural difficulties, except for the prosocial behaviour scale, where lower scores indicate greater difficulties. Each SDQ score was categorised as either "normal" or "borderline/abnormal" based on binary cut-off scores recommended by Goodman: total behavioural difficulties (≥14), emotional symptoms (≥4), peer relationship problems (≥3), conduct problems (≥3), hyperactivity (≥6), and prosocial behaviours (≤5). Missing data for child’s sex recorded at birth n = 2.

**Table S4:** Mean scores for total difficulties and each SDQ sub-scale, stratified by child sex and age (n = 646).

| **Child behavioural problems** |  |  | **Mean (SD)** | | |  |
| --- | --- | --- | --- | --- | --- | --- |
|  | **All** | **Boys** | **Girls** | **2 years** | **3 years** | **4-8 years** |
| Total behavioural difficulties | 10.5 (5.6) | 10.9 (5.6) | 10.0 (5.6) | 9.9 (4.7) | 10.4 (5.4) | 11.6 (7.5) |
| Emotional symptoms | 1.7 (1.8) | 1.6 (1.7) | 1.9 (1.9) | 1.3 (1.4) | 1.7 (1.7) | 1.7 (2.5) |
| Peer relationship problems | 1.7 (1.7) | 1.8 (1.8) | 1.6 (1.7) | 1.9 (2.8) | 1.5 (1.6) | 1.7 (2.0) |
| Conduct problems | 2.2 (1.8) | 2.3 (1.9) | 2.1 (1.8) | 2.1 (1.7) | 2.4 (1.8) | 2.3 (2.2) |
| Hyperactivity/inattention | 4.7 (2.8) | 5.1 (2.5) | 4.3 (2.6) | 4.6 (2.3) | 4.8 (2.5) | 4.8 (3.2) |
| Prosocial behaviour | 7.4 (2.0) | 6.9 (2.1) | 7.9 (1.8) | 7.2 (2.1) | 7.5 (1.7) | 7.9 (2.5) |

**Table S5.** Association between preconception folic acid intake and borderline/abnormal behavioural problems, stratified by child’s age and sex.

|  | **Total behavioural difficulties** | **Emotional problems** | **Peer relationship problems** | **Conduct problems** | **Hyperactivity/ inattention** | **Prosocial behaviours** |
| --- | --- | --- | --- | --- | --- | --- |
| Preconception folic acid intake (*Ref. = No*) |  | At age 2-3 years (n = 517), RR (95% CI) ^a^ | | |  |  |
|  | 1.08 (0.73, 1.57) | 0.75 (0.43, 1.28) | **0.64 (0.46, 0.89)** | 1.10 (0.87, 1.40) | 1.03 (0.79, 1.33) | 0.91 (0.62, 1.33) |
|  |  | At age 4-8 years (n = 129) | |  |  |  |
|  | 0.99 (0.54, 1.83) | 0.97 (0.47, 1.99) | 0.92 (0.44, 1.93) | 0.64 (0.35, 1.16) | 0.50 (0.29, 1.01) | 0.25 (0.09, 1.05) |
|  |  | Boys only (n = 335) | | |  |  |
|  | 1.14 (0.73, 1.78) | 0.82 (0.44, 1.56) | **0.49 (0.33, 0.75)** | 0.93 (0.69, 1.25) | 0.50 (0.89, 1.54) | 0.69 (0.46, 1.03) |
|  |  | Girls only (n = 309) | | |  |  |
|  | 0.96 (0.65, 1.23) | 0.77 (0.47, 1.25) | 0.70 (0.46, 1.05) | 0.92 (0.66, 1.27) | 0.81 (0.57, 1.15) | 0.53 (0.24, 1.14) |

^a^ Adjusted relative risk (aRR) with 95% CI were estimated using Poisson regression with robust standard error variance (0 = Normal, 1 = Borderline/abnormal behaviours), with "Normal behaviours" as the reference group. Adjusted for maternal characteristics (maternal age at child’s birth, area of residence, level of education, marital status, ability to manage on income, number of children, perceived psychological distress, and intimate partner violence), BMI, maternal vegetable intake, physical activity, smoking, alcohol intake.

**Legend**

- Pink nodes represent confounders
- Blue nodes represent covariates

**Figure S1:** Directed acyclic graph showing the relationship between folic acid intake and borderline/abnormal child behavioural problems.

 **Figure S2:** Relationship between preconception folic acid intake and behavioural problems in children aged 2–8 years (n = 646). Adjusted for maternal characteristics (maternal age at child’s birth, residence, education, marital status, ability to manage on income, intimate partner violence, number of children, pre-pregnancy BMI, maternal vegetable intake, physical activity, smoking, alcohol consumption, child’s sex and age.
